# Exposure of hospitalised pregnant women to plasticizers contained in medical devices

**DOI:** 10.1186/s12905-017-0398-7

**Published:** 2017-06-20

**Authors:** Cécile Marie, Sebti Hamlaoui, Lise Bernard, Daniel Bourdeaux, Valérie Sautou, Didier Lémery, Françoise Vendittelli, Marie-Pierre Sauvant-Rochat

**Affiliations:** 1 Axe TGI-PEPRADE, Institut Pascal, Sigma Clermont, CNRS, Université Clermont Auvergne, 63001 Clermont-Ferrand, France; 2 Service biostatistique, Pôle Santé Publique, CHU de Clermont-Ferrand, 63003 Clermont-Ferrand, France; 3 Pôle Pharmacie, CHU Clermont-Ferrand, ICCF, SIGMA Clermont, CNRS, Université Clermont Auvergne, F-63000 Clermont–Ferrand, France; 4 Pôle Gynécologie-obstétrique, CHU de Clermont-Ferrand, 63003 Clermont-Ferrand, France; 5 AUDIPOG (Association des Utilisateurs de Dossiers informatisés en Pédiatrie, Obstétrique et Gynécologie), RTH Laennec Medical University, 69372 Lyon, France; 6 Département Santé Publique et Environnement, Faculté de Pharmacie, Université Clermont Auvergne, 63001 Clermont-Ferrand, France

**Keywords:** Alternative plasticizers, In-utero exposure, Medical devices, Obstetrics;phthalates

## Abstract

**Background:**

Medical devices (MDs) in polyvinyl chloride (PVC) are not a well-known source of exposure to plasticizers, in particular during pregnancy. Because of its toxicity, the di-(2-ethylhexyl) phthalate (DEHP) has been replaced by other plasticizers such as di (isononyl)-cyclohexane-1,2-dicarboxilic acid (DINCH), tri-octyltrimellitate (TOTM) and di-(isononyl) phthalate (DiNP). Our study aimed to quantify the plasticizers (DEHP and alternative plasticizers) contained in PVC medical devices used for hospitalised pregnant women and to describe which these MDs had been used (type, number, duration of exposure).

**Methods:**

The plasticizers contained in the MDs used for daily care in the Obstetrics Department of a French University Hospital were extracted from PVC (after contact with a chloroform solution), identified and quantified by gas-chromatography-mass-spectrometry analysis. A total of 168 pregnant women hospitalised in the Obstetrics Department with at least one catheter were included in the observational study. The median number of MDs containing plasticizers used and the daily duration of exposure to the MDs were compared in three groups of pregnant women: “Pathology group” (women hospitalised for an obstetric disorder who did not give birth during this hospitalisation; *n* = 52), “Pathology and delivery group” (hospitalised for an obstetric disorder and who gave birth during this stay; *n* = 23) and “Delivery group” (admitted for planned or spontaneous delivery without obstetric disorder; *n* = 93).

**Results:**

DiNP, TOTM and DINCH were the predominant plasticizers contained in the MDs at an amount of 29 to 36 g per 100 g of PVC. Women in the “Pathology group” (preterm labour or other pathology) were exposed to a median number of two MDs containing TOTM and one MD containing DiNP, fewer than those in the “Pathology and delivery group” (*p* < 0.05). Women in the “Pathology group” had a median exposure of 3.4 h/day to MDs containing DiNP and 8.2 h/day to MDs containing TOTM, longer than those in the “Delivery group” (*p* < 0.01).

**Conclusions:**

Our study shows that the medical management of pregnant women in a hospital setting entails exposure to MDs containing alternative plasticizers (DiNP, TOTM and DINCH).

**Electronic supplementary material:**

The online version of this article (doi:10.1186/s12905-017-0398-7) contains supplementary material, which is available to authorized users.

## Background

Polyvinyl chloride (PVC) plasticizers are widely present in the environment. These compounds are mainly used to soften PVC and to improve the flexibility of a broad range of objects including food packaging, toys, flooring, shower curtains, and cables. In particular, they enter into the composition of medical devices (MD) made with PVC such as tubing and medical gloves [1–4].

Until 2010, the most commonly used plasticizer in MDs was di-(2-ethylhexyl) phthalate (DEHP), which can account for 30 to 40% of the weight of plastics for medical use [5]. As they have no chemical bond with plastic materials, phthalates are easily released into the environment and DEHP can be released from MDs [6–8]. During intravenous infusion, release from MDs is enhanced by factors such as temperature, duration of use, surface contact, infusion rate and contact with lipophilic substances [6, 9–12].

Phthalates are the subject of increasing concern owing to their effects on human health [13–16]. Proof of their toxicity [17–20] has led European regulators to restrict the use of certain phthalates in numerous products. MDs containing phthalates classified as class 1 or 2 carcinogenic and mutagenic chemical substances and/or toxic for reproduction [DEHP, di-butyl-phthalate (DBP), benzyl-butyl-phthalate (BBP)] must be labelled as such. For at-risk populations (children, pregnant or breastfeeding women), the use of these substances must be justified [21]. In France, since July 2015, the use of MDs with DEHP is banned in paediatric, neonatal and maternity departments [22].

To replace these phthalates in MDs, manufacturers have incorporated other plasticizers such as acetyltri-n-butyl citrate (ATBC), di-isononyl-1,2-cyclohexane-dicarboxylate (DINCH), trioctyl trimellitate (TOTM), di-(ethylhexyl)-terephthalate (DEHT), di-(ethylhexyl)-adipate (DEHA) and diisononyl-phthalate (DiNP). However, there is still scant documented evidence about the potential ability of these new plasticizers to migrate from PVC nor about their toxicity [6].

There has been little research about exposure of pregnant women to plasticizers in a medical setting. However, during pregnancy, when the vital organs are still developing and its metabolism is immature, the fœtus is particularly vulnerable [23]. In utero exposure to phthalates (DEHP and DBP in particular) has been associated with an increased risk of foetal masculinisation disorder [15, 24, 25] and preterm birth [26, 27]. Some authors have imputed use of MDs during pregnancy to an increase in phthalate exposure [28–31]. However, the risk of contamination of biological samples and the small sample sizes in the published studies make it impossible to conclude whether intravenous treatments contribute to increased exposure to phthalates during pregnancy and delivery. In addition, the authors did not state what types of MD were used (bags, tubing, etc.), their composition (with or without PVC), their insertion time, nor the drugs that may have been in contact with the MDs [28–31]. Finally, to our knowledge no study has assessed exposure to alternative DEHP-free plasticizers during the medical management of pregnancy.

The principal objective of this study, was to detect and quantify the plasticizers (DEHP and alternative plasticizers) contained in the PVC devices used for hospitalised pregnant women. The secondary objective was to describe among a sample population of hospitalised pregnant women which MDs containing the plasticizers TOTM, DiNP, DINCH, DEHT and DEHP had been used (type, number, duration of exposure).

## Methods

### Analysis of medical devices (MDs)

#### Materials

Pertinent information for the MDs available for daily care in the Obstetrics Department of the University Hospital of Clermont-Ferrand, France are summarized in Table 1. In all, 15 different MDs were used: 8 with PVC (3 infusion sets, 4 extension tubes and 1 flow regulator) and 7 without PVC (4 catheters, 1 syringe for electric syringe pump and 2 urinary catheters). The MDs which have undergone a qualitative and quantitative analysis of the plasticizers are only those which may contain phthalates and/or other plasticizers in their formulation, namely the MDs based on PVC. The other MDs were made of elastomeric materials (polyurethane and silicone) or polyolefin (polypropylene) which are free of plasticizers (Table 1).

**Table 1:** Characteristics of medical devices used for pregnant women included in the study and GC-MS analyses results

Information on the packaging and/or the technical sheets of the MDs					GC-MS Analysis
Medical device (Manufacturer)	References	Batch number	Material	Mention “with DEHP” or “with phthalates”	PVC plasticizers (% of plasticizers: g of plasticizers / 100 g of PVC)^a^
Catheters					
Venflon™ pro safety (BD Medical Systems)	393228; 393229; 393224; 393226	4114335P47	PU	No	Not analyzed^b^
Surshield Versatus™ (Terumo)	SR + DS1845PX	2110058	PU	No	Not analyzed^b^
Insyte™ Autoguard™ (BD Medical Systems)	381934; 381944	3225842	PU	No	Not analyzed^b^
Epidural catheterization set (Teleflex)	JC05400B	71F14J1867	PU	No	Not analyzed^b^
Infusion Sets					
Infusion set perfusend (Sendal)	A64	03501	PVC	No	DiNP (36.39%).
Multi access infusion set (Doran Int)	Edelvaiss-2	231501Q	PVC/TOTM	No	TOTM (34.99%), DEHP (0.01%), DEHT (0.13%).
Epidural infusion set (Smiths medical)	21–7039-24	2020–01	PVC/TOTM	DEHP <0.2%	TOTM (34.93%), DEHP (1.19%).
Extension tubes (for electric syringe pump)					
Extension tube (Cair LGL)	PB311 8 M	14H27-T	PE/PVC	No	TOTM (30.87%), DEHP (0.02%), DEHT (0.95%).
Extension tube (Cair LGL)	PB311 5 M	14G30-T	PE/PVC	No	TOTM (28.79%), DEHP (0.02%), DEHT (0.43%).
Extension tube (Cair LGL)	PB310 5 M	14F02-T	PE/PVC	No	TOTM (30.01%), DEHP (0.01%), DEHT (0.35%).
Extension line with 3 way stopcock (Cair LGL)	PES3301M	14I06-T	PE/PVC	No	TOTM (30.03%), DEHP (0.02%), DEHT (0.48%).
Flow Regulator					
Flow regulator (Cair LGL)	SSDF050	14H25-T	PVC	No	DINCH (33.50%), DEHP (0.01%).
*S*YRINGES *(for electric syringe pump)*					
BD plastipak syringe (BD Medical Systems)	300865	1407212	PP	No	Not analyzed^b^
Urinary catheters					
Intermittent urinary catheter (Coloplast)	275140	4227421	PU	No^c^	Not analyzed^b^
Indwelling urinary catheter (Teleflex)	170605–000140	144E06	Silicone	No	Not analyzed^b^

^a^The quantity of each plasticizer contained in MDs was expressed in g of plasticizer for 100 g of PVC which corresponds to the percentage (%) of plasticizers contained in the PVC of MDs

^b^The MDs which have undergone a GC-MS analysis are only those which may contain phthalates/other plasticizers in their formulation, namely the MDs based on PVC. The other MDs made of unplasticized biomaterial [elastomeric materials (polyurethane and silicone) or polyolefin (polypropylene)] were not analyzed because they are free of plasticizers

^c^Limits of detection of 10 to 40 μg/g of product

Abbreviations: *DEHP* di-(2-ethylhexyl) phthalate, *DEHT* di-(ethylhexyl)-terephthalate, *DINCH* di(isononyl)-cyclohexane-1,2-dicarboxilic acid, *DiNP* di-(isononyl) phthalate; *GC-MS* gas chromatography-mass spectrometry, *MDs* medical devices, *PE* Polyethylene, *PP* Polypropylene, *PU* Polyurethane, *PVC* polyvinyl chloride, *TOTM* tri-octyltrimellitate

#### Methods

According to the European regulation, suppliers of MDs must mention on the packaging the presence of DEHP when it exceeds 0.1% by mass of the plasticized material [21]. There are no other recommendations for alternative plasticizers. For our study, the manufacturer’s technical data sheet for each MD was consulted to establish the composition of the device (PVC or other material), to check whether the presence of phthalates or DEHP and/or the presence of alternative plasticizers (DiNP, TOTM, DEHT, DEHA, etc.) were mentioned. Only one PVC device (an infusion set for epidural analgesia) specified the presence of DEHP (<0.2%). Only two technical data sheets (one for a multi-access infusion set and the other for an epidural infusion set) specified the PVC plasticizer used (TOTM). The type of phthalates and/or other plasticizers in the other PVC devices was not mentioned in the manufacturer’s technical data sheets (Table 1).

The composition of the plasticizers (DEHP, DiNP, TOTM, DEHA, etc.) in the PVC devices was assessed by gas chromatography-mass spectrometry (GC/MS) as described by Bourdeaux et al. (2016) [32]. The plasticizers were extracted from MDs as follows. A minimum of 10 mg PVC was cut with a scalpel, carefully weighed and placed in a 25 mL flask filled with chloroform containing 2 μg/mL of benzyl-butyl-phthalate (BBP) as internal standard. Extraction was made by simply soaking the sample in the BBP chloroform solution at ambient temperature. After one hour of contact (the optimal extraction time determined by Bernard et al. (2015) [33]), the solution was homogenized and 1 mL was removed and placed in a chromatography vial for GC/MS analysis. The chromatographic analysis was performed with a chromatograph coupled to a Clarus 500 mass spectrometer (Perkin Elmer, USA). The column used was a 5 Accent Optima (30 m × 0.25 μm 0.25 mmID) (Macherey-Nagel, Germany). The oven temperature was increased from 200 to 300 °C at a rate of 20 °C/min to reach a plateau of 300 °C for 7 min. The temperature of the injector was increased to 300 °C and that of the transfer and the source electron impact line maintained at 200 °C. The ionization energy source was 70 eV. The carrier gas flow (helium) was maintained at 1.2 mL/min and the leakage flow (split) at 20 ml/min. One μL of each sample was injected. Calibration curves were performed with five calibration points from 0.1 to 25 μg/mL of each plasticizer and 2 μg/mL of BBP to obtain coefficients of determination r^2^ > 0.999 for all plasticizers. The method had good precision and accuracy with coefficients of variation not exceeding 10% for any of the plasticizers. The lower limits of quantification used were 0.1 μg/mL for DEHA, ATBC, DEHT and DEHP, 0.25 μg/mL for DINCH, 0.5 μg/mL for TOTM and 1.5 μg/mL for DINP [32]. The mean recovery values for the 7 plasticizers were as follows: ATBC 100.7%, DEHA 106.7%, DEHP 100.8%, DINCH 102.2%, DEHT 94.9%, DINP 84.5%, and TOTM 89.0% (the mean intra-day and inter-day relative standard deviation were below 10%) [32].

The quantity of each plasticizer contained in MDs was expressed in g of plasticizer for 100 g of PVC which corresponds to the percentage (%) of plasticizers contained in the PVC of MDs.

### Assessment of women exposure to medical devices (MDs) containing PVC plasticizers

#### Materials

The study population was made up of women admitted to the University Hospital of Clermont-Ferrand, France, for pathological pregnancy and/or delivery. Pregnant women who had at least one peripheral venous catheter inserted during the hospital stay were eligible for inclusion in the study. Non-inclusion criteria were: women hospitalised other than in the obstetrics department, women without peripheral catheter inserted, women admitted for medical termination of pregnancy or in utero foetal death and unwillingness of the midwives of the obstetrics department or the pregnant women to take part in the study.

In accordance with the French human research ethics law, this study was exempt from approval by the French Institutional Review because our database contained no nominative data, and the project was not an interventional research study. The medical database was submitted to the French Data Protection Authority (CNIL: Commission National de l’Informatique et des Libertés) as report number 1268114.

#### Methods

A descriptive study was performed. Because of organisational constraints on the medical teams, the study was conducted over two periods, from 7 March to 20 April 2013, and from 1 March to 31 May 2014. The same MDs were used in the department during both periods of investigation. The MDs were not grouped by the type but by the predominant plasticizer present in the device. We made this choice because a same type of MD, such as infusion tubes, for example, can contain different plasticizers depending on the manufacturer. A woman was considered as exposed to an MD containing a plasticizer if she came into contact at least once with an MD containing the additive during hospitalisation. Over the whole study period, 1318 deliveries were made and 559 women were admitted for pathological pregnancies defined by the presence of obstetric disorder during their pregnancy (such as preterm labour, gestational arterial hypertension, etc.). Our final study sample comprised 168 women.

**Fig. 1:**
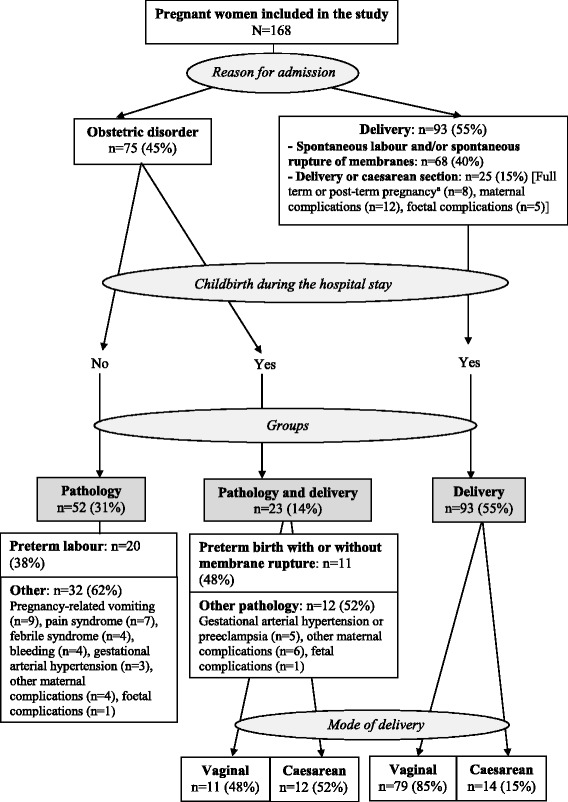
Reasons for hospital admission of the women enrolled in the study. ^a^Full-term is defined as pregnancy lasting 41 completed weeks and post-term pregnancy is defined as pregnancy lasting 42 completed weeks

The women taking part in the study were divided into three groups for the analysis. The “Pathology group” comprised women hospitalised for an obstetric disorder who did not give birth during their stay. For this group, the reason for hospital admission could be variable (i.e., preterm labour without delivery, pregnancy-related vomiting, bleeding, gestational arterial hypertension, etc.). The “Pathology and delivery group” was composed of women admitted for an obstetric disorder and who gave birth during the hospital stay (i.e, reason for hospital admission were preterm labour with or without membrane rupture, gestational arterial hypertension, foetal complication, etc.). The “Delivery group” was made up of women admitted for planned or spontaneous delivery and who had not been hospitalised for obstetric disorder before childbirth. The reasons for hospitalisation and the division of the participants into three groups are shown in Fig. 1.

#### Data collection

Data useful for the study were recorded prospectively by a designated investigator in collaboration with the midwives. The investigator was present daily in the department to analyse prescriptions. Sociodemographic details and medical data, including the reason for hospital admission, were retrieved from computerised obstetric files kept by physicians and midwives in the department. For each type of MD (catheter, infusion tube, extension tube, syringe for electric syringe pump, urinary catheter), the product reference, the manufacturer, the date and time of the beginning and end of use were recorded.

#### Data analysis

The qualitative variables were compared among the three groups by a Pearson’s Chi-square or a Fisher’s exact test, as appropriate. For a given plasticizer, the total duration of exposure corresponds to the overall duration of use of the MD containing the plasticizer during the whole hospital stay. The total duration of exposure was divided by the length of hospital stay (in days) to obtain the daily duration of exposure (in hours per day). The number of MDs used and the daily duration of exposure to MDs containing the plasticizers were compared among the three groups by a Kruskal-Wallis nonparametric test (because of the non-normal distribution of these variables).

Significance was defined by *p* < 0.05. Statistical analyses were performed with R statistical software, version 2.15.2 (R Development Core Team, Vienna, Austria, 2012).

## Results

### Analysis of medical devices (MDs)

The results of the GC-MS analyses of MDs in PVC are presented in Table 1. Trioctyl trimellitate (TOTM) was the predominantly detected plasticizer in multi-access infusion sets (35% or g of TOTM/100 g of PVC), extension tubes (30% or g of TOTM/100 g of PVC) and epidural infusion sets (35% or g of TOTM/100 g of PVC). Diisononyl-phthalate (DiNP) was the predominantly PVC plasticizer detected in simple infusion sets (37% or g of DiNP/100 g of PVC). Di-isononyl-1,2-cyclohexane-dicarboxylate (DINCH) was the main plasticizer detected in flow regulators (33.5% or g of DINCH/100 g of PVC). Di-(2-ethylhexyl) phthalate (DEHP) was present in MDs with a majority content of TOTM or DINCH, most often in “trace” form (< 0.1% or g of DEHP/100 g of PVC), and in a greater proportion for epidural infusion sets (1.2% or g of DEHP/100 g of PVC). Di-(ethylhexyl)-terephthalate (DEHT) was present at rates ranging from 0.1 to 0.95% or g of DEHT/100 g of PVC in MDs having a majority content of TOTM. In MDs containing DiNP, no other plasticizer was detected (Table 1).

### Exposure of pregnant women to medical devices (MDs) containing PVC plasticizers

One hundred and sixty-eight women were included the study. The mean age of the women was 28.7 +/− 5.7 years. The other obstetric characteristics before hospitalisation are not given (Additional file 1). The maternal obstetric characteristics at the time of admission are shown in Table 2. The mean length of hospital stay of all the women was 6.4 +/− 4.4 days.

**Table 2:** Maternal obstetric characteristics at the time of hospitalisation

Studied groups	Gestational age	Hospitalisation duration	Onset of labour (*n* = 116)			Mode of delivery (*n* = 116)	
			Spontaneous	Induction^c^	Caesarean^d^	Vaginal	Caesarean^e^
	[M +/− SD]^a^	[M +/− SD]^b^	n (%)	n (%)	n (%)	n (%)	n (%)
Total, *n* = 168	[34.4 +/− 7.8]	[6,4 +/− 4.4]	72 (62.1)	30 (25.9)	14 (12.1)	90 (77.6)	26 (22.4)
Pathology, *n* = 52	[26.2 +/− 8.3]	[4.7 +/− 3.0]	-	-	-	-	-
PL^f^, *n* = 20	[30.4 +/− 2.5]	[5.6 +/− 3.3]	-	-	-	-	-
Other^g^, *n* = 32	[23.6 +/− 9.6]	[4.2 +/− 2.8]	-	-	-	-	-
Delivery, *n* = 93	[39.3 +/− 2.4]	[6,0 +/− 1.5]	59 (63.4)	25 (26.9)	9 (9.7)	79 (84.9)	14 (15.1)
Vaginal, *n* = 79	[39.5 +/− 1.7]	[5.8 +/− 1.4]	57 (72.2)	22 (27.8)	0	79 (100)	0
Caesarean, *n* = 14	[37.9 +/− 4.5]	[7.1 +/− 1.7]	2 (14.3)	3 (21.4)	9 (64.3)	0	14 (100)
Pathology and delivery, *n* = 23	[33.0 +/− 4.5]	[12.0 +/− 8.7]	13 (56.5)	5 (21.7)	5 (20.8)	11 (47.8)	12 (52.2)
PL-PRM^h^, *n* = 11	[30.6 +/− 3.7]	[8.8 +/− 4.4]	9 (81.8)	0	2 (18.2)	6 (54.5)	5 (45.5)
Other^i^, *n* = 12	[35.3 +/− 4.0]	[14.9 +/− 10.7]	4 (33.3)	5 (41.7)	3 (25.0)	5 (41.7)	7 (58.3)

^a^Gestational age is expressed in weeks [mean +/− standard deviation]

^b^Hospitalisation duration is expressed in days [mean +/− standard deviation]

^c^Reasons for induction: rupture of membranes >24 h without labour (*n* = 13), full-term (pregnancy lasting 41 completed weeks) or post-term pregnancy (pregnancy lasting 42 completed weeks) (*n* = 8), gestational arterial hypertension or preeclampsia (*n* = 4), suspected foetal macrosomia (*n* = 2), other maternal disease (*n* = 3)

^d^Caesarean before and during labour

^e^Reasons for caesarean before labour: foetal complications (*n* = 4), maternal complications (*n* = 5), maternal/foetal complications (*n* = 5)

^f^Preterm labour

^f^Other obstetrical pathologies: pregnancy-related vomiting (*n* = 9), bleeding (*n* = 4), gestational arterial hypertension (*n* = 3), pain syndrome (*n* = 7), febrile syndrome (*n* = 4), preterm rupture of membranes or suspicion (*n* = 2), convulsion (*n* = 1), cervical incompetence (*n* = 1) and foetal complication (twin-to-twin transfusion syndrome, *n* = 1)

^g^Preterm labour (*n* = 7) and/or preterm rupture of membranes (*n* = 4)

^h^Other obstetrical pathologies: bleeding (*n* = 2), gestational arterial hypertension or preeclampsia (*n* = 4) pain syndrome (*n* = 1), febrile syndrome (*n* = 1), cholestasis (*n* = 1), suspected neurological transient ischemic attack (*n* = 1) and foetal complication (intrauterine growth restriction, *n* = 1)

The “Pathology group” accounted for 31% of the women, of whom 38% were admitted for a preterm labour and 62% for another pathology. The “Pathology and delivery group” represented 14% of the women, of whom 48% were admitted for preterm birth with or without membrane rupture and 52% for another pathology. The “Delivery group” made up 55% of the women in the study (Fig. 1).

Ninety percent of the women during their hospital stay were exposed to PVC devices at least once (Table 3). Consequently, they were potentially exposed to three main plasticizers (TOTM, DiNP and DINCH). During their hospital stay, 74% of the women were exposed at least once to a simple infusion set predominantly containing DiNP, 73% at least once to an MD predominantly containing TOTM (multi-access infusion set, epidural infusion set or extension tube) and 4% at least once to the flow regulator predominantly containing DINCH. Women in the “Pathology group” were significantly less exposed to MDs containing DiNP (48%) and TOTM (40%) than those in the other two groups (*p* < 0.001). Exposure to MDs containing DiNP was most frequent in women in the “Pathology and delivery group” (91%), and exposure to an MD containing TOTM was most frequent in the “Delivery group” (89%). Only the women in the “Pathology group” were exposed to at least one MD containing DINCH (11.5%). These women had all been admitted for pregnancy-related vomiting episodes (Table 3).

**Table 3:** Exposure of pregnant women to medical devices containing PVC plasticizers during their hospital stay

	Total *N* = 168 (%)	Pathology group *n* = 52 (%)	Pathology and delivery group *n* = 23 (%)	Delivery group *n* = 93 (%)	*p*-value
Exposure at least to one MD with PVC	151 (90)	35 (67%)	23 (100)	93 (100)	-
Exposure at least to one MD with PVC according to the predominantly plasticizer^a^					
DEHP	0	0	0	0	-
DiNP^b^	124 (74)	25 (48)	21 (91)	78 (84)	< 0.001
TOTM^c^	123 (73)	21 (40)	19 (83)	83 (89)	< 0.001
DINCH^d^	6 (4)	6 (11.5)	0	0	-

^a^Data based on gas chromatography-mass spectrometry analysis; during their hospital stay, women could be exposed to different MDs containing various plasticizers

^b^Type of MD containing DiNP: simple infusion set

^c^Type of MDs containing TOTM: multi-access infusion set, epidural infusion set and extension tubes

^d^Type of MD containing DINCH: flow regulator; all women exposed to at least one MD containing DINCH had been admitted for pregnancy-related vomiting episodes

Abbreviations: *DEHP* di-(2-ethylhexyl) phthalate, *DINCH* di(isononyl)-cyclohexane-1,2-dicarboxilic acid, *DiNP* di-(isononyl) phthalate, *MD* medical device, *PVC* polyvinyl chloride, *TOTM* tri-octyltrimellitate

**Fig. 2:**
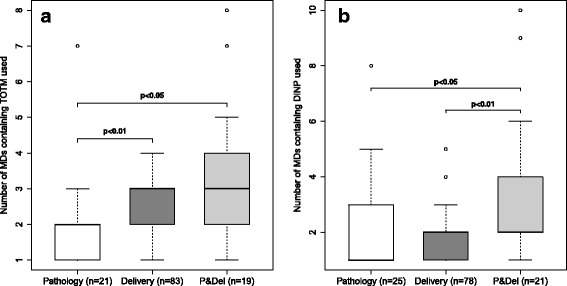
Number of PVC devices containing TOTM or DiNP used during the women’s hospital stay. Women exposed at least once to MDs containing TOTM (**a**) (*n* = 123) or DiNP (**b**) (*n* = 124). Abbreviations: DiNP, di-(isononyl) phthalate; MD, medical device; P&Del, “Pathology and delivery group”; PVC, polyvinyl chloride; TOTM, tri-octyltrimellitate

Among the women exposed at least once to MDs containing TOTM, those in the “Pathology group” were exposed to a median number of two MDs and those in the other two groups to a median number of three (*p* < 0.05) (Fig. 2). Among the women exposed at least once to MDs containing DiNP, those in the “Pathology group” were exposed to a median number of one MD and those in the other two groups to a median number of two (statistically significant difference between the “Pathology group” and “Pathology and delivery group”, *p* < 0.05) (Fig. 2).

**Fig. 3:**
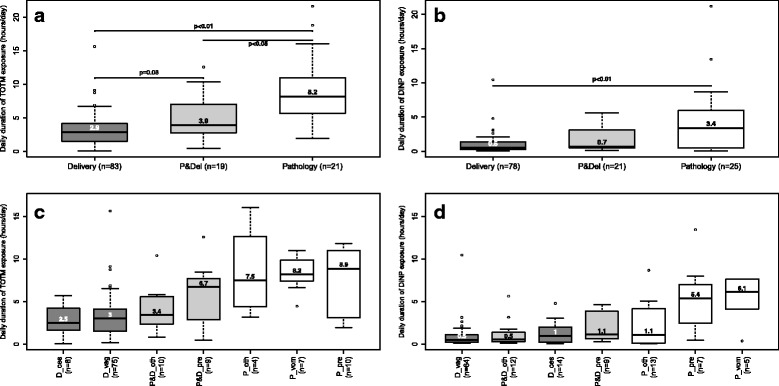
Daily duration of exposure to MDs containing TOTM and DiNP during the women’s hospital stay. Women exposed at least once to MDs containing TOTM (**a** and **c**) (*n* = 123) or DiNP (**b** and **d**) (*n* = 124). Abbreviations: *D_ces* “Delivery group” and Caesarean section; D_vag, “Delivery group” and vaginal delivery; *DiNP* di-(isononyl) phthalate; *MD* medical device; *P_oth* “Pathology group” and other pathology; *P_pre* “Pathology group” and preterm labour; *P_vom* “Pathology group” and pregnancy-related vomiting; *P&D_oth* “Pathology and delivery group” and other pathology; *P&D_pre* “Pathology and delivery group” and preterm labour; *P&Del* “Pathology and delivery group”; *PVC* polyvinyl chloride; *TOTM* tri-octyltrimellitate

Women in the “Pathology group” had statistically longer median daily exposure to TOTM (8.2 h/day) and DiNP (3.4 h/day) than those in the “Delivery group” (TOTM: 2.9 h/day; DiNP: 0.5 h/day) (*p* < 0.01) (Fig. 3). The daily median exposure time of the women in the “Pathology and delivery group” was midway between that of the other two groups. Women in the “Pathology group” who had been admitted for preterm labour and pregnancy-related vomiting had the longest exposure to MDs containing plasticizers. Finally, the women in this hospitalised group for vomiting were the only ones exposed to DINCH, for a median time of 6.9 h/day (Fig. 3).

Results about the type of drugs administered by intravenous route, and thus in contact with the MDs containing plasticizers are not shown (Additional files 2 and 3).

## Discussion

### DEHP and alternative plasticizers contained in medical devices

According to the information provided on the packaging and in the technical data sheets of the MDs, none of the devices used in our study contained DEHP as the predominant plasticizer. Hence, in 2013–2014, the Obstetrics Department of the University Hospital of Clermont-Ferrand followed the recommendations of the EU Regulation (2008), which requires that the DEHP content of MDs be clearly indicated [21]. However, one of the MDs in our study, an epidural infusion set, was labelled as containing less than 0.2% of DEHP, which is above the threshold of <0.1% authorized by the REACH regulation [34]. Our analysis showed the presence of DEHP, most often in trace form, in MDs containing predominantly TOTM or DINCH. The threshold of 0.1% authorised by REACH was in most cases respected except for the epidural infusion set (1.2% or g of DEHP/100 g of PVC). This finding is consistent with those of other experimental studies which discovered that PVC MDs presented as DEHP-free (particularly those containing TOTM) were in fact contaminated with DEHP and DEHT, at concentrations above the level authorised for DEHP by REACH regulations [35, 36]. This contamination, which is due to the presence of impurities (ortho- and para-phthalic acid) during synthesis of the plasticizers, carries a non-zero risk of exposure for pregnant women. Exposure to these doses of DEHP lower than 0.1% should be incorporated into the assessment of risk involved in the use of plasticizers in the composition of MDs.

Four alternative plasticizers were identified and quantified in the MDs used in our obstetric department (TOTM, DiNP, DINCH and DEHT). Most of the PVC devices used in our department contained TOTM, as in another study made in France [36]. Overall these plasticizers represented from 29 to 40 g/100 g of PVC in the MDs studied. The alternative plasticizers are often found in large quantities because their plasticizing properties are less effective than those of DEHP. For example, compared to DEHP, TOTM has a substitution factor of 1.17 [37].

### Exposure to medical devices containing alternative plasticizers

To our knowledge, this study is the first to assess concurrently the amounts of alternative plasticizers contained in MDs and the clinical use of these MDs for pregnant women admitted to an obstetrics department. All of the women who gave birth at hospital (the “Delivery and Pathology group” and “Delivery group”) were exposed to MDs containing TOTM or DiNP, compared to less than half of those in the “Pathology group”. The number of MDs containing TOTM was statistically lower in the “Pathology group” but the daily duration of exposure was statistically longer over the whole hospital stay for these women. The same trend was observed with DiNP. These findings warrant comment. Although the women hospitalised for an obstetric disorder had considerable exposure to the three plasticizers (median time, respectively, of 8.2, 6.9 and 3.4 h/day for TOTM, DINCH and DiNP), this factor is not the one that best reflects the risk of contamination. The number of MDs used for each women is often a better indicator because each MDs can release a certain amount of plasticizers after its use. In fact, the kinetics of migration of plasticizers shows that these plasticizers are mainly released in the 24 h after the first contact between the PVC device and its contents (the amount of plasticizer released remains generally stable over time) [6]. Hence, each time a PVC device is changed, women are exposed to potentially higher doses.

The conclusions to be drawn from these findings are even more important when we consider that our study concerned only one hospital stay. Women with a pathological pregnancy can be hospitalised, and thus exposed, several times until delivery. Almost half the women in our study (44%) had already been admitted at least once since the beginning of their pregnancy. Gestational age at the time of hospitalisation is also an important factor to take into account. In general, for most teratogens, exposure at the beginning of pregnancy, when the vital organs are still developing, will not have the same consequences for the fœtus compared to a later exposure [38]. For the phthalates, several studies have reported an increased risk of shortened anogenital distance and cardiac malformation after exposure during the first trimester of pregnancy [24, 25, 39]. By comparison, exposure to phthalates in the third trimester has been associated with a greater risk of preterm birth and adverse cognitive and behavioral outcomes in children [26, 27, 40]. Fetal Outcomes will also depend on the type of chemicals and the levels of exposure.

### Plasticizer-related health risks for pregnant women and fœtus

The three main plasticizers detected in our study, DiNP (which like DEHP belongs to the class of phthalates), TOTM (which belongs to the class of trimellitate esters) and DINCH (which belongs to that of cyclohexanes) should be considered separately owing to differences in their physicochemical properties and toxicological profiles.

TOTM is released in far smaller amounts than DEHP in the same conditions of use, principally as a result of its higher molecular weight and greater steric hindrance and its lower solubility in water [6, 7, 41]. Bernard et al. (2015) [6] showed that only 0.2% of the total initial amount of the TOTM present in the tubing migrated into a simulant (migration tests conducted at 40 °C in a temperature-controlled chamber for 24 h). In the few experimental studies available, the no observed adverse effect level (NOAEL) relative to the development of the male reproductive system was markedly higher for TOTM (100 mg/kg/day) than for DEHP (5 mg/kg/day) [2, 42]. The toxic effects of TOTM act on the liver, increasing its weight and the peroxisomes in the hepatocytes, trigger oestrogenic activity, decrease the number of spermatozoids and spermatocytes in male rats and can lead to developmental anomalies such as testicular ectopy and areolar retention [2, 42–45].

Little is known about the potential ability of DiNP to migrate from MDs. A study from the SCENHIR report (2014) [2] showed that in lipophilic media leaching of DiNP is similar to that of DEHP. The physicochemical properties of the two compounds are also comparable, with DiNP having a slightly higher molecular weight and a slightly lower solubility in water [37]. DiNP is banned in the composition of toys and childcare articles that can be placed in the mouth [46] because it can leach into the saliva [47]. Animal toxicity studies have shown its carcinogenic effects on the liver, as the result of the proliferation of hepatic peroxisomes, and on the kidney. However, these findings cannot be extrapolated to humans because the effects of peroxisome proliferation in the liver of rodents are seen as species-specific [48–50]. The potentially reprotoxic effects of DiNP are similar to those of DEHP, but at higher doses: the highest NOAEL is 15 mg/kg/day for DiNP as against 5 for DEHP [2]. In rodents, prenatal exposure to DiNP was associated with anti-androgenic effects such as impairment of germinal and Leydig cells, a decrease in testicular production of testosterone, testicular atrophy, reduced anogenital distance and reduced sperm motility [18, 51–54]. DiNP is the only plasticizer whose effects on humans have been studied. In utero exposure to DiNP was associated with an increased risk of smaller anogenital distance in newborn boys [24].

The potential migration of DINCH from MDs is not clearly established. One study showed that the rate of DINCH migration from enteral nutrition tubing was 5-fold lower than that of DEHP after 24 h of contact [55] while another reported a similar rate of 1/8 of the initial content for the two over the same period [6]. Furthermore two studies carried out by the manufacturer of DINCH (BASF) concluded that the product had no impact on reproduction and established a NOAEL of 1000 mg/kg/day [1, 2]. However, in one of these studies anogenital distance was statistically significantly reduced, by 7 to 8%, in male and female rats exposed to high doses during gestation. The effect was not considered biologically relevant because of the small extent of the decrease, the difference in distance between the sexes (in females, the distance is generally smaller) and the absence of any other effect on the reproductive system. Another chronic toxicity study established a NOAEL of 40 mg/kg/day in males on the basis of the hepatic and renal effects observed in rats [1, 2].

Thus, current evidence is insufficient to accurately assess the health risks of exposure to these replacement plasticizers. Either we lack information on migration rates (principally for DiNP and DINCH), or on toxicity (principally for TOTM and DINCH).

Another important factor to be taken into consideration when assessing migration of plasticizers from PVC is the type of drug that comes in contact with the MDs. For example, release of plasticizers contained in MDs is enhanced by contact with lipophilic substances [9, 12, 40, 56]. Data on drugs in contact with the MDs are shown in the supplementary materials (Additional files 2 and 3).

### Limitations

Our study has several limitations. Firstly, our analytical study quantifies only the amounts of plasticizers contained in MDs and not the amounts migrating from PVC. Further studies are needed to specify the migration of these alternative plasticizers. These migration data, combined with our results on the use of MDs in clinical practice, could give an estimate of the doses of exposure among pregnant women. This approach needs to be validated by the determination of biomarkers. For DiNP and DINCH, which metabolise rapidly in the organism and are largely excreted in the urine, oxidized metabolites measured in urine are reliable biomarkers [57–59]. In contrast, for TOTM, which accumulates in the tissues and is eliminated more slowly, mainly in the stools [60], measuring metabolites in urine is not suitable. However, biomarker assays would have given an estimate of total exposure during hospitalisation and not just exposure to MDs. Exposure to plasticizers in the hospital is not limited to contact with MDs, and contamination can occur via inhalation of phthalates in PVC flooring material [61, 62], consumption of food prepared with PVC gloves [63] and/or contained and heated in plastic recipients [64], and by skin contact with medical gloves [4]. The presence of these other sources of exposure does not bias our findings regarding the objectives of our study (focused on the theoretical exposure due to the use of MDs). Nevertheless these multiples sources of exposure in medical settings should be further investigated in order to determine their specific contribution to the total exposure.

Our study did not assess exposure to MDs throughout pregnancy but only during a hospital stay. However, analysis of the results from the three groups studied (“Pathology”, “Pathology and delivery”, and “Delivery”), allowed us to study different stages in pregnancy and to highlight the importance of exposure to plasticizers in a hospital setting. Moreover, our study is not representative of the overall population of hospitalised pregnant women since only those with at least one catheter were eligible. Consequently, the proportion of women exposed to MDs with PVC was overestimated. However, this bias of selection was limited because in our department a catheter is inserted at admission for a large proportion of women. Our patient sample is not representative of the overall Auvergne area since the pregnant women admitted to the University Hospital of Clermont-Ferrand, which is the only level III maternity department of the region, represented a high risk population. Consequently, it can be assumed that the use of MDs is more common in this maternity department than in the level I and II units of our region. Finally, the purchase of MDs is regulated by the conditions of public contracts between the hospital and the manufacturers. These conditions are subject to change and in the future different MDs and hence different PVC plasticizers may be used in the department where our study was conducted.

## Conclusion

Our study shows that the medical management of pregnant women in a hospital setting entails exposure to MDs containing alternative plasticizers of DEHP such as DiNP, TOTM and DINCH. The next step of our study will be to accurately quantify the plasticizers released from PVC to drug solutions (by using migration tests) and therefore the doses of exposure of pregnant women. Nevertheless, given the potential toxic effects of DINP, TOTM and DINCH, prevention measures to limit the exposure of the mother and fœtus could in the meantime be taken, such as a reasonable use that avoids unnecessary practices or too frequent changing of tubing during critical periods of foetal development.

## Additional files

Additional file 1: Table S1. Maternal obstetric characteristics before hospitalisation. (DOC 48 kb)

Additional file 2: Drugs administered intravenously during the women’s hospital stay. According to group (A); according to the type of plasticizer in contact with the drug (B). Abbreviations: DINCH, di (isononyl)-cyclohexane-1,2-dicarboxilic acid; DiNP, di-(isononyl) phthalate; ESP, electric syringe pump; TOTM, tri-octyltrimellitate. The term “epidural analgesics” refers to levobupivacaine and sufentanil; “antibiotics” to amoxicillin (*n* = 29), ceftriaxone (*n* = 15), clindamycin (*n* = 3), cefixime (*n* = 2), aztreonam (*n* = 1) and ertapenem (*n* = 1); “antiemetics” to chlorpromazine (*n* = 5), metoclopramide (*n* = 2), and ondansetron (*n* = 1); “other (ESP)” to nicardipine, esomeprazole, diazepam, potassium chloride and gluconate, hydroxyzine, hydroxyethyl starch and iron; and “other” to dinoprostone, nicardipine and morphine. (PDF 32 kb)

Additional file 3: Drugs administered intravenously during the women’s hospital stay according to their lipophilicity. Log P is the octanol–water partitioning coefficient (log K_ow_) of the drug in its uncharged form. The higher the log P, the more lipophilic the drug. Log *P* values are based on the following sources: phloroglucinol [http://www.chemicalland21.com/lifescience/phar/1,3,5-TRIHDROXY%20BENZENE.htm], dinoprostone, ertapenem, esomeprazole, iron and nalbuphine (DrugBank database, version 4.2 [http://www.drugbank.ca/]), other drugs (EPI Suite™ Kow-Win program [http://www.epa.gov/oppt/exposure/pubs/episuitedl.htm]; Fick et al., 2010). Abbreviations: cef, cefixime; chl, chlorpromazine; cli, clindamycin; dia, diazepam; din, dinoprostone; ert, ertapenem; eso, esomeprazole; hyd, hydroxyzine; met, metoclopramide; mor, morphine; nic, nicardipine; ond, ondansetron. (PDF 8 kb)

## Abbreviations

ATBC: Acetyltri-n-butyl citrate
BBP: Benzyl-butyl-phthalate
CNIL: Commission National de l’Informatique et des Libertés
DBP: Di-butyl-phthalate
DEHA: Di-(ethylhexyl)-adipate
DEHP: Di-(2-ethylhexyl) phthalate
DEHT: Di-(ethylhexyl)-terephthalate
DINCH: Di (isononyl)-cyclohexane-1,2-dicarboxilic acid
DiNP: Di-(isononyl) phthalate
GC/MS: Gas chromatography-mass spectrometry
MD: Medical device
NOAEL: No observed adverse effect level
PVC: Polyvinyl chloride
TOTM: Tri-octyltrimellitate

## References

[1] Babich MA (2010). Review of exposure and toxicity data for phthalate substitutes.

[2] Scientific Committee on Emerging and Newly-Identified Health Risks (SCENIHR). Opinion on the Safety Of Medical Devices Containing DEHP-Plasticized PVC or Other Plasticizers on Neonates and Other Groups Possibly at Risk (2014 update). SCENIHR, 2014. http://ec.europa.eu/health/scientific_committees/emerging/docs/scenihr_o_047.pdf. Accessed 8 Jan 2016. 10.1016/j.yrtph.2016.01.013 26854686

[3] Schettler T (2006). Human exposure to phthalates via consumer products. Int J Androl.

[4] Wormuth M, Scheringer M, Vollenweider M, Hungerbühler K (2006). What are the sources of exposure to eight frequently used phthalic acid esters in Europeans?. Risk Anal.

[5] Council of Europe. Materials based on plasticised poly(vinyl chloride) for containers for aqueous solutions for intravenous infusion. In: European Pharmacopoeia 5.0, vol. 1. Council of Europe. 2004:296–298.

[6] Bernard L, Cueff R, Breysse C, Décaudin B, Sautou V, Armed Study Group (2015). Migrability of PVC plasticizers from medical devices into a simulant of infused solutions. Int J Pharm.

[7] Kambia K, Dine T, Azar R, Gressier B, Luyckx M, Brunet C (2001). Comparative study of the leachability of di(2-ethylhexyl) phthalate and tri(2-ethylhexyl) trimellitate from haemodialysis tubing. Int J Pharm.

[8] Strac IV, Pušić M, Gajski G, Garaj-Vrhovac V (2013). Presence of phthalate esters in intravenous solution evaluated using gas chromatography-mass spectrometry method. J Appl Toxicol.

[9] Bagel S, Dessaigne B, Bourdeaux D, Boyer A, Bouteloup C, Bazin JE (2011). Influence of lipid type on bis (2-ethylhexyl)phthalate (DEHP) leaching from infusion line sets in parenteral nutrition. J Parenter Enter Nutr.

[10] Gotardo MA, Monteiro M (2005). Migration of diethylhexyl phthalate from PVC bags into intravenous cyclosporine solutions. J Pharm Biomed Anal.

[11] Kambia K, Dine T, Gressier B, Bah S, Germe AF, Luyckx M (2003). Evaluation of childhood exposure to di(2-ethylhexyl) phthalate from perfusion kits during long-term parenteral nutrition. Int J Pharm.

[12] Rose RJ, Priston MJ, Rigby-Jones AE, Sneyd JR (2012). The effect of temperature on di(2-ethylhexyl) phthalate leaching from PVC infusion sets exposed to lipid emulsions. Anaesthesia.

[13] Jurewicz J, Polańska K, Hanke W (2013). Exposure to widespread environmental toxicants and children's cognitive development and behavioral problems. Int J Occup Med Environ Health.

[14] López-Carrillo L, Hernández-Ramírez RU, Calafat AM, Torres-Sánchez L, Galván-Portillo M, Needham LL (2010). Exposure to phthalates and breast cancer risk in northern Mexico. Environ Health Perspect.

[15] Marie C, Vendittelli F, Sauvant-Rochat MP (2015). Obstetrical outcomes and biomarkers to assess exposure to phthalates: a review. Environ Int.

[16] Swan SH (2008). Environmental phthalate exposure in relation to reproductive outcomes and other health endpoints in humans. Environ Res.

[17] Foster PM, Mylchreest E, Gaido KW, Sar M (2001). Effects of phthalate esters on the developing reproductive tract of male rats. Hum Reprod Update.

[18] Gray LE, Ostby J, Furr J, Price M, Veeramachaneni DN, Parks L (2000). Perinatal exposure to the phthalates DEHP, BBP, and DINP, but not DEP, DMP, or DOTP, alters sexual differentiation of the male rat. Toxicol Sci.

[19] Parks LG, Ostby JS, Lambright CR (2000). The plasticizer diethylhexyl phthalate induces malformations by decreasing fetal testosterone synthesis during sexual differentiation in the male rat. Toxicol Sci.

[20] Wilson VS, Lambright C, Furr J, Abbott BD, Klinefelter GR, Barlow NJ (2004). Phthalate ester-induced gubernacular lesions are associated with reduced insl3 gene expression in the fetal rat testis. Toxicol Lett.

[21] European Union. Regulation (EC) No 1272/2008 of the European Parliament and of the Council of 16 December 2008 on classification, labelling and packaging of substances and mixtures, amending and repealing Directives 67/548/EEC and 1999/45/EC, and amending Regulation (EC) No 1907/2006; 2008.

[22] République Française; Assemblée Nationale. Amendement du 27 mars 2015 de la Loi n° 2012–1442 du 24 décembre 2012 visant à la suspension de la fabrication, de l'importation, de l'exportation et de la mise sur le marché de tout conditionnement à vocation alimentaire contenant du bisphénol A;2015.

[23] Dimanti-Kandarakis E, Bourguignon JP, Giudice LC, Hauser R, Prins GS, Soto AM (2009). Endocrine-disrupting chemicals: an endocrine society scientific statement. Endocr Rev.

[24] Bornehag CG, Carlstedt F, Jönsson BA, Lindh CH, Jensen TK, Bodin A (2015). Prenatal phthalate exposures and anogenital distance in Swedish boys. Environ Health Perspect.

[25] Swan SH, Sathyanarayana S, Barrett ES, Janssen S, Liu F, Nguyen RH (2015). TIDES study team. First trimester phthalate exposure and anogenital distance in newborns. Hum Reprod.

[26] Ferguson KK, McElrath TF, Meeker JD (2014). Environmental phthalate exposure and preterm birth. JAMA Pediatr.

[27] Meeker JD, Hu H, Cantonwine DE, Lamadrid-Figueroa H, Calafat AM, Ettinger AS (2009). Urinary phthalate metabolites in relation to preterm birth in Mexico city. Environ Health Perspect.

[28] Vandentorren S, Zeman F, Morin L, Sarter H, Bidondo ML, Oleko A (2011). Bisphenol-a and phthalates contamination of urine samples by catheters in the Elfe pilot study: implications for large-scale biomonitoring studies. Environ Res.

[29] Yan X, Calafat A, Lashley S, Smulian J, Ananth C, Barr D (2009). Phthalates biomarker identification and exposure estimates in a population of pregnant women. Hum Ecol Risk Assess.

[30] Huang Y, Li J, Garcia JM, Lin H, Wang Y, Yan P (2014). Phthalate levels in cord blood are associated with preterm delivery and fetal growth parameters in Chinese women. PLoS One.

[31] Huang PC, Kuo PL, Guo YL, Liao PC, Lee CC (2007). Associations between urinary phthalate monoesters and thyroid hormones in pregnant women. Hum Reprod.

[32] Bourdeaux D, Yessaad M, Chennell P, Larbre V, Eljezi T, Bernard L (2016). Armed study group. Analysis of PVC plasticizers in medical devices and infused solutions by GC-MS. J Pharm Biomed Anal.

[33] Bernard L, Cueff R, Bourdeaux D, Breysse C, Sautou V, Armed Study Group (2015). Analysis of plasticizers in poly(vinyl chloride) medical devices for infusion and artificial nutrition: comparison and optimization of the extraction procedures, a pre-migration test step. Anal Bioanal Chem.

[34] European Union. Regulation (EC) No 1907/2006 of the European Parliament and of The Council of 18 December 2006 concerning the Registration, Evaluation, Authorisation and Restriction of Chemicals (REACH), establishing a European Chemicals Agency, amending Directive 1999/45/EC and repealing Council Regulation (EEC) No 793/93 and Commission Regulation (EC) No 1488/94 as well as Council Directive 76/769/EEC and Commission Directives 91/155/EEC, 93/67/EEC, 93/105/EC and 2000/21/EC; 2006.

[35] Genay S, Luciani C, Décaudin B, Kambia N, Dine T, Azaroual N (2011). Experimental study on infusion devices containing polyvinyl chloride: to what extent are they di(2-ethylhexyl)phthalate-free?. Int J Pharm.

[36] Gimeno P, Thomas S, Bousquet C, Maggio AF, Civade C, Brenier C, et al. Identification and quantification of 14 phthalates and 5 non-phthalate plasticizers in PVC medical devices by GC-MS. J 2014. 949:99–108. 10.1016/j.jchromb.2013.12.037 24480330

[37] Bernard L, Décaudin B, Lecoeur M, Richard D, Bourdeaux D, Cueff R (2014). Armed study Group. Analytical methods for the determination of DEHP plasticizer alternatives present in medical devices: a review. Talanta.

[38] Holmes LB (2011). Human teratogens: update 2010. Birth Defects Res A Clin Mol Teratol.

[39] Wang C, Zhan Y, Wang F, Li H, Xie L, Liu B (2015). Parental occupational exposures to endocrine disruptors and the risk of simple isolated congenital heart defects. Pediatr Cardiol.

[40] Ejaredar M, Nyanza EC, Ten Eycke K, Dewey D (2015). Phthalate exposure and childrens neurodevelopment: a systematic review. Environ Res.

[41] Ito R, Miura N, Iguchi H, Nakamura H, Ushiro M, Wakui N (2008). Determination of tris(2-ethylhexyl)trimellitate released from PVC tube by LC-MS/MS. Int J Pharm.

[42] United States Consumer Product Safety Commission (CPSC). Chronic hazard advisory panel on phthalates and phthalate alternatives. Final report. Bethesda: United States CPSC; 2014. https://www.cpsc.gov/PageFiles/169876/CHAP-REPORT-FINAL.pdf. Accessed 7 Jan 2016.

[43] Hodgson JR (1987). Results of peroxisome induction studies on tri(2-ethylhexyl)trimellitate and 2-ethylhexanol. Toxicol Ind Health.

[44] Huntingdon Life Sciences. TEHTM study for effects on embryo-fetal and pre- and post-natal development in CD rat by oral gavage administration. Sanitized Version. Huntingdon Life Sciences, Ltd; 2002.

[45] ter Veld MG, Schouten B, Louisse J, van Es DS, van der Saag PT, Rietjens IM (2006). Estrogenic potency of food-packaging-associated plasticizers and antioxidants as detected in ERalpha and ERbeta reporter gene cell lines. J Agric Food Chem.

[46] European Union. Directive 2005/84/EC of the European Parliament and of the council of. Amending for the 22nd Time Council Directive 76/769/EEC on the Approximation of the Laws, Regulations and Administrative Provisions of the Member States Relating to Restrictions on the Marketing and Use of Certain Dangerous Substances and Preparations (phthalates in toys and childcare articles). 14 December 2005:2005.

[47] Babich MA, Chen SB, Greene MA, Kiss CT, Porter WK, Smith TP (2004). Risk assessment of oral exposure to diisononyl phthalate from children's products. Regul Toxicol Pharmacol.

[48] European Chemical Agency (ECHA). Evaluation of new scientific evidence concerning DINP and DIDP in relation to entry 52 of Annex XVII to REACH Regulation (EC) No 1907/2006: Final review report. ECHA: Helsinki, Finland, 2013. http://echa.europa.eu/documents/10162/31b4067e-de40-4044-93e8-9c9ff1960715. Accessed 7 Jan 2016.

[49] Lington AW, Bird MG, Plutnick RT, Stubblefield WA, Scala RA (1997). Chronic toxicity and carcinogenic evaluation of diisononyl phthalate in rats. Fundam Appl Toxicol.

[50] Shaw D, Lee R, Roberts RA (2002). Species differences in response to the phthalate plasticizer monoisononylphthalate (MINP) in vitro: a comparison of rat and human hepatocytes. Arch Toxicol.

[51] Boberg J, Christiansen S, Axelstad M, Kledal TS, Vinggaard AM, Dalgaard M (2011). Reproductive and behavioral effects of diisononyl phthalate (DINP) in perinatally exposed rats. Reprod Toxicol.

[52] Clewell RA, Thomas A, Willson G, Creasy DM, Andersen ME (2013). A dose response study to assess effects after dietary administration of diisononyl phthalate (DINP) in gestation and lactation on male rat sexual development. Reprod Toxicol.

[53] Hannas BR, Lambright CS, Furr J, Howdeshell KL, Wilson VS, Gray LE (2011). Dose-response assessment of fetal testosterone production and gene expression levels in rat testes following in utero exposure to diethylhexyl phthalate, diisobutyl phthalate, diisoheptyl phthalate, and diisononyl phthalate. Toxicol Sci.

[54] Li L, Bu T, Su H, Chen Z, Liang Y, Zhang G (2015). In utero exposure to diisononyl phthalate caused testicular dysgenesis of rat fetal testis. Toxicol Lett.

[55] Welle F, Wolz G, Franz R (2005). Migration of plasticizers from PVC tubes into enteral feeding solutions. Pharma International.

[56] Loff S, Kabs F, Witt K, Sartoris J, Mandl B, Niessen KH (2000). Polyvinylchloride infusion lines expose infants to large amounts of toxic plasticizers. J Pediatr Surg.

[57] Silva MJ, Reidy JA, Preau JL, Needham LL, Calafat AM (2006). Oxidative metabolites of diisononyl phthalate as biomarkers for human exposure assessment. Environ Health Perspect.

[58] Silva MJ, Jia T, Samandar E, Preau JL, Calafat AM (2013). Environmental exposure to the plasticizer 1,2-cyclohexane dicarboxylic acid, diisononyl ester (DINCH) in U.S. adults (2000-2012). Environ Res.

[59] Dereumeaux C, Saoudi A, Pecheux M, Berat B, de Crouy-Chanel P, Zaros C (2016). Biomarkers of exposure to environmental contaminants in French pregnant women from the Elfe cohort in 2011. Environ Int.

[60] Martis L, Freid E, Woods E (1987). Tissue distribution and excretion of tri-(2-ethylhexyl)trimellitate in rats. J Toxicol Environ Health.

[61] Bornehag CG, Lundgren B, Weschler CJ, Sigsgaard T, Hagerhed-Engman L, Sundell J (2005). Phthalates in indoor dust and their association with building characteristics. Environ Health Perspect.

[62] Carlstedt F, Jönsson BA, Bornehag CG (2013). PVC flooring is related to human uptake of phthalates in infants. Indoor Air.

[63] Tsumura Y, Ishimitsu S, Saito I, Sakai H, Kobayashi Y, Tonogai Y (2001). Eleven phthalate esters and di(2-ethylhexyl) adipate in one-week duplicate diet samples obtained from hospitals and their estimated daily intake. Food Addit Contam.

[64] Cirillo T, Fasano E, Esposito F, Montuori P, Amodio CR (2013). Di(2-ethylhexyl)phthalate (DEHP) and di-n-butylphthalate (DBP) exposure through diet in hospital patients. Food Chem Toxicol.

