# Supplementary material for: Exposure of hospitalised pregnant women to plasticizers contained in medical devices
Source: BMC Womens Health. 2017 Jun 20;17:45. doi: 10.1186/s12905-017-0398-7 (PMC5480197; doi:10.1186/s12905-017-0398-7)
Supplement: Supplementary file 1 — Maternal obstetric characteristics before hospitalisation. (DOC 48 kb) [file 12905_2017_398_MOESM1_ESM.doc]

**Appendix** **1** Maternal obstetric characteristics before hospitalisation

| **Studied groups** | **Maternal age** | **Previous pregnancy**  ^b^ | **Previous parity** ^c^ | **Twin pregnancy** | **Previous hospitalisation** ^d^ |
| --- | --- | --- | --- | --- | --- |
|  | [M +/- SD] ^a^ | n (%) | n (%) | n (%) | n (%) |
| **Total,** n=168 | [28.7 +/- 5.7] | 103 (61.3) | 87 (51.8) | 14 (8.3) | 73 (43.5) |
| **Pathology**, n=52 | [27.8 +/- 5.7] | 34 (65.4) | 24 (46.2) | 5 (9.6) | 24 (46.2) |
| **PL** ^e^, n=20 | [27.6 +/- 5.2] | 11 (55.0) | 10 (50.0) | 2 (10.0) | 11 (55.0) |
| **Other** ^f^, n=32 | [28.0 +/- 6.0] | 23 (71.9) | 14 (43.8) | 3 (9.4) | 13 (40.6) |
| **Delivery**, n=93 | [29.0 +/- 5.7] | 51 (54.8) | 50 (53.8) | 3 (3.2) | 31 (33.3) |
| **Vaginal**, n=79 | [28.9 +/- 5.4] | 43 (54.4) | 46 (58.2) | 3 (3.8) | 22 (27.8) |
| **Caesarean**, n=14 | [29.7 +/- 7.5] | 8 (57.1) | 4 (28.6) | 0 | 9 (64.3) |
| **Pathology and delivery**, n=23 | [29.5 +/- 5.8] | 18 (78.3) | 13 (56.5) | 6 (26.1) | 18 (78.3) |
| **PL-PRM** ^g^, n=11 | [27.3 +/- 4.9] | 8 (72.7) | 7 (63.6) | 3 (27.3) | 10 (90.9) |
| **Other** ^h^, n=12 | [31.6 +/- 6.0] | 10 (83.3) | 6 (50.0) | 3 (25.0) | 8 (66.7) |

^a^ Maternal age is expressed in year [mean +/- standard deviation].

^b^ Previous pregnancy ≥ 1.

^c^ Previous parity ≥ 1.

^d^ Previous hospitalisation during this pregnancy (≥ 1).

^e^ Preterm labour.

^f^ Other obstetrical pathologies: pregnancy-related vomiting (n=9), bleeding (n=4), gestational arterial hypertension (n=3), pain syndrome (n=7), febrile syndrome (n=4), preterm rupture of membranes or suspicion (n=2), convulsion (n=1), cervical incompetence (n=1) and foetal complication (twin-to-twin transfusion syndrome, n=1).

^g^ Preterm labour (n=7) and/or preterm rupture of membranes (n=4).

^h^ Other obstetrical pathologies: bleeding (n=2), gestational arterial hypertension or preeclampsia (n=4) pain syndrome (n=1), febrile syndrome (n=1), cholestasis (n=1), suspected neurological transient ischemic attack (n=1) and foetal complication (intrauterine growth restriction, n=1).
