# Supplementary figures and images for: Exposure of hospitalised pregnant women to plasticizers contained in medical devices
Source: BMC Womens Health. 2017 Jun 20;17:45. doi: 10.1186/s12905-017-0398-7 (PMC5480197; doi:10.1186/s12905-017-0398-7)

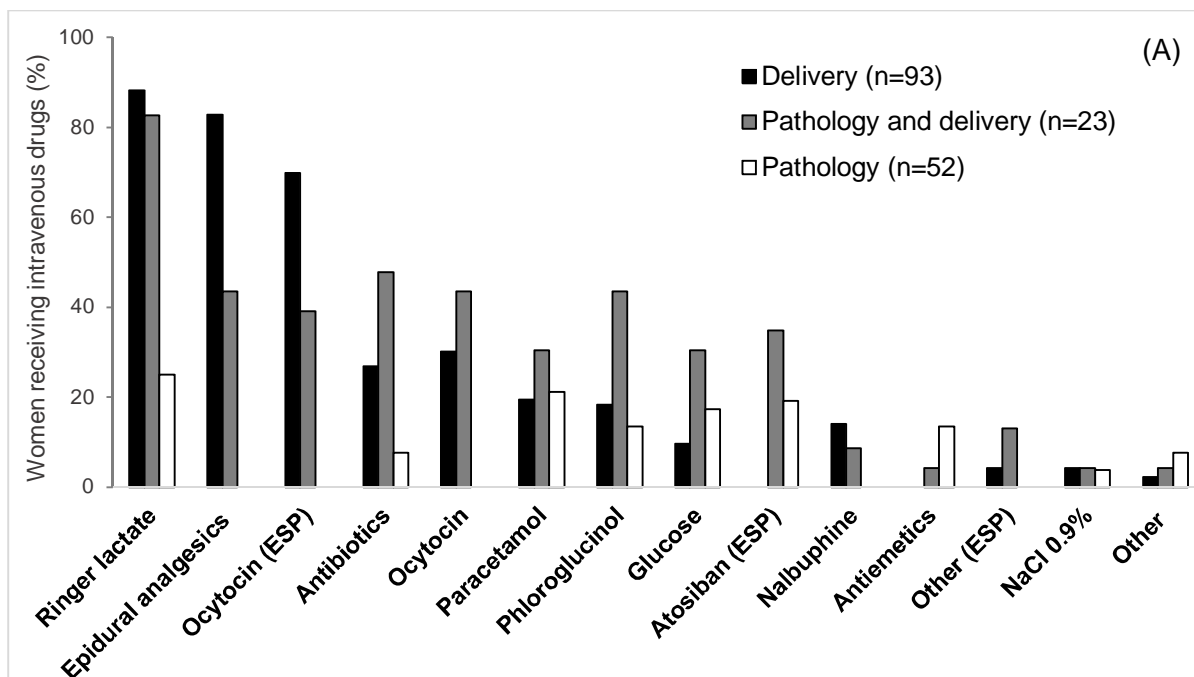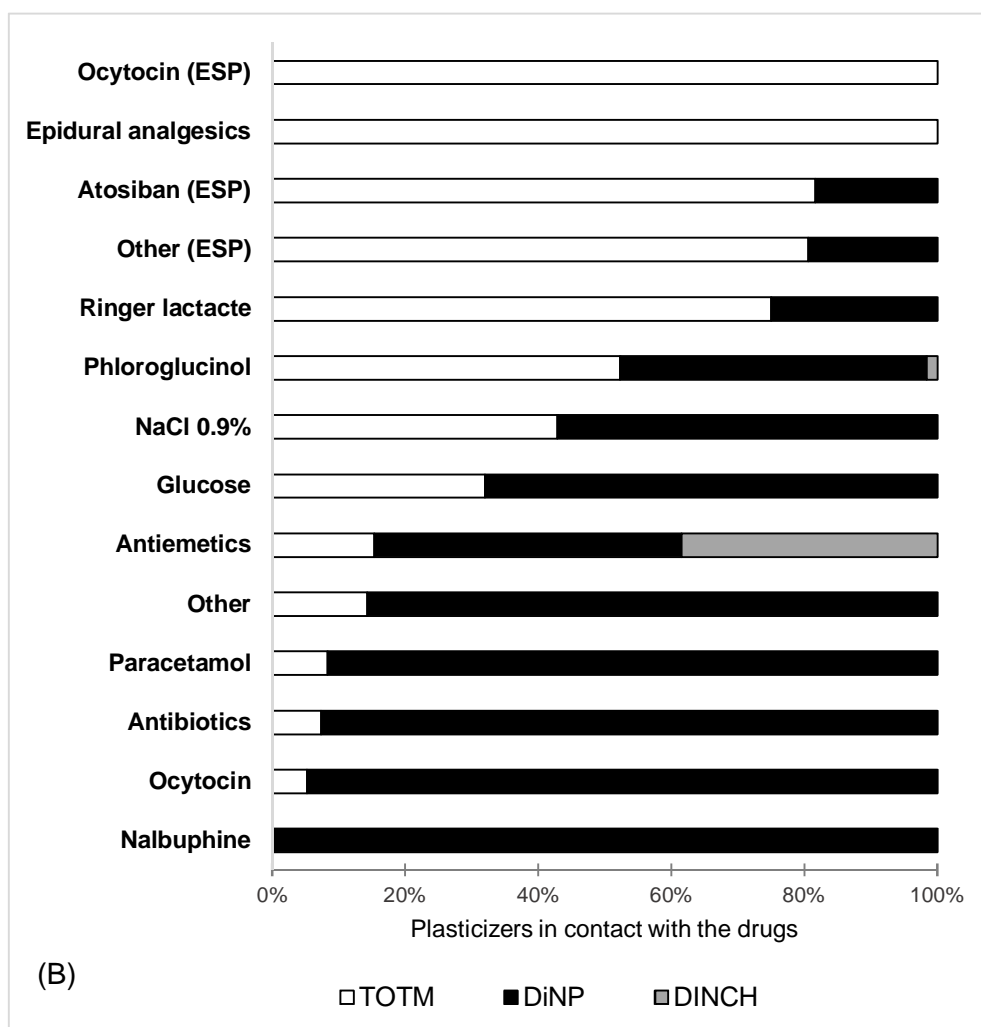

Supplement: Supplementary file 2 — Drugs administered intravenously during the women’s hospital stay. According to group (A); according to the type of plasticizer in contact with the drug (B). Abbreviations: DINCH, di (isononyl)-cyclohexane-1,2-dicarboxilic acid; DiNP, di-(isononyl) phthalate; ESP, electric syringe pump; TOTM, tri-octyltrimellitate. The term “epidural analgesics” refers to levobupivacaine and sufentanil; “antibiotics” to amoxicillin (n = 29), ceftriaxone (n = 15), clindamycin (n = 3), cefixime (n = 2), aztreonam (n = 1) and ertapenem (n = 1); “antiemetics” to chlorpromazine (n = 5), metoclopramide (n = 2), and ondansetron (n = 1); “other (ESP)” to nicardipine, esomeprazole, diazepam, potassium chloride and gluconate, hydroxyzine, hydroxyethyl starch and iron; and “other” to dinoprostone, nicardipine and morphine. (PDF 32 kb) [file 12905_2017_398_MOESM2_ESM.pdf]

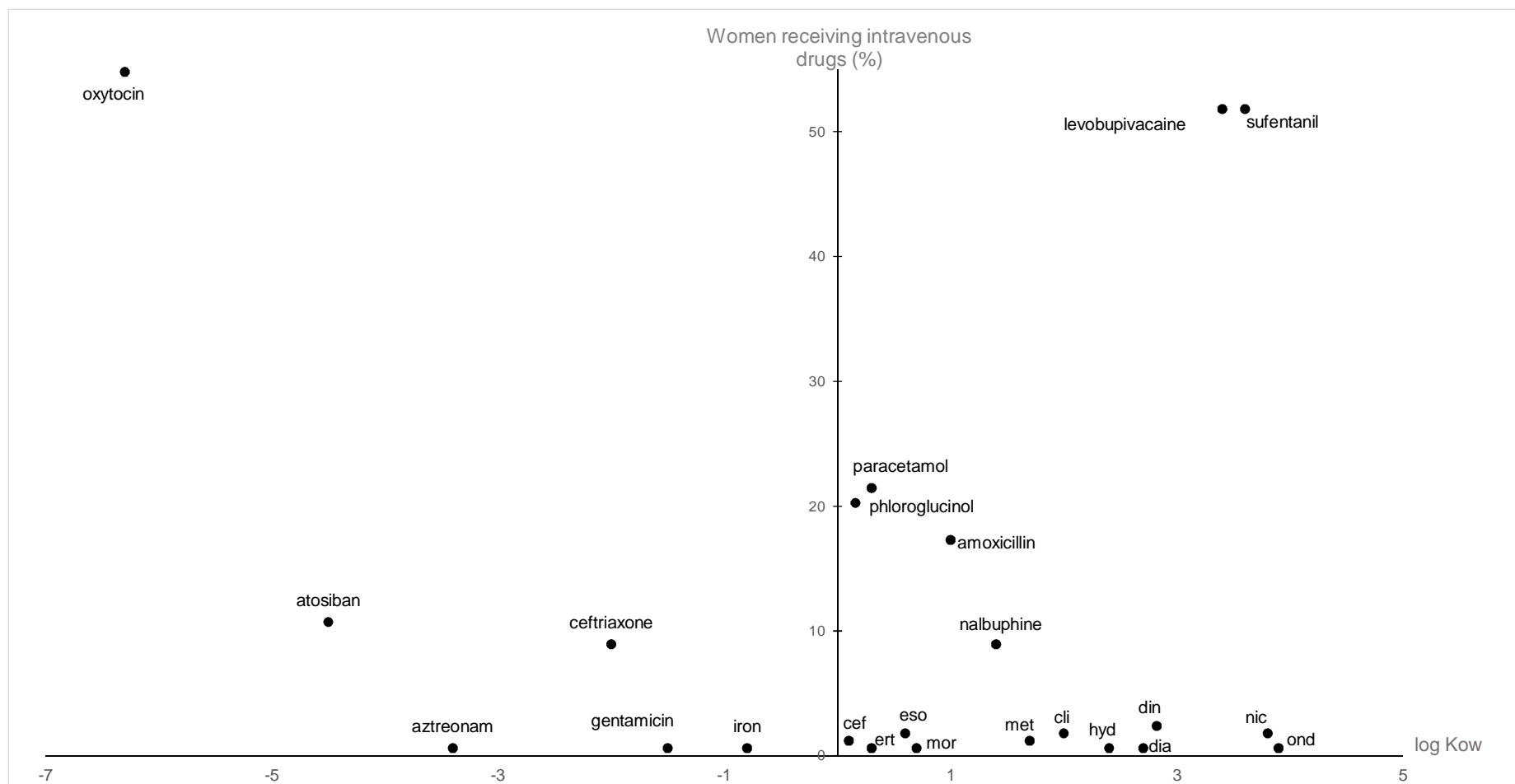

Supplement: Supplementary file 3 — Drugs administered intravenously during the women’s hospital stay according to their lipophilicity. Log P is the octanol–water partitioning coefficient (log Kow) of the drug in its uncharged form. The higher the log P, the more lipophilic the drug. Log P values are based on the following sources: phloroglucinol [http://www.chemicalland21.com/lifescience/phar/1,3,5-TRIHDROXY%20BENZENE.htm], dinoprostone, ertapenem, esomeprazole, iron and nalbuphine (DrugBank database, version 4.2 [http://www.drugbank.ca/]), other drugs (EPI Suite™ Kow-Win program [http://www.epa.gov/oppt/exposure/pubs/episuitedl.htm]; Fick et al., 2010). Abbreviations: cef, cefixime; chl, chlorpromazine; cli, clindamycin; dia, diazepam; din, dinoprostone; ert, ertapenem; eso, esomeprazole; hyd, hydroxyzine; met, metoclopramide; mor, morphine; nic, nicardipine; ond, ondansetron. (PDF 8 kb) [file 12905_2017_398_MOESM3_ESM.pdf]
